# Tracking anthropogenic nitrogen-compound sources of surface and groundwater in southwestern Nile Delta: hydrochemical, environmental isotopes, and modeling approach

**DOI:** 10.1007/s11356-022-23536-1

**Published:** 2022-10-25

**Authors:** Rasha Hussien, Mona Ahmed, Aly Islam Aly

**Affiliations:** Nuclear and Radiological Safety Research Center, Egyptian Atomic Energy Authority, Cairo, Egypt

**Keywords:** Nitrate contamination, Environmental stable isotopes, Nitrogen isotope content, MODFLOW-MT3D contaminant transport, Southwestern Nile Delta, Egypt

## Abstract

This research aims to assign the specific and potential sources that control migration and transformation mechanisms of ammonium/nitrate contaminants of surface and groundwater systems in the southwestern Nile Delta, Egypt. To achieve that, an integration of hydrogeochemistry, multiple environmental stable isotopes (δ^2^H-H_2_O, δ^18^O-H_2_O, δ^15^N-NH_4_, and δ^15^N-NO_3_) coupled with three-dimensional nitrogen transport numerical model (MODFLOW-MT3D) was done. A set of representative water samples (20 canals and drainage water) and 14 groundwater samples were collected and analyzed for physical, chemical, and stable isotope analysis. NH_4_^+^ and NO_3_^−^ concentrations in surface water samples varied from 0.29 to 124 mg/l and 0.52 to 39.67 mg/l, respectively. For groundwater samples, NH_4_^+^ and NO_3_^−^ concentrations varied from 0.21 to 1.75 mg/l and 0.33 to 32.8 mg/l, respectively. Total risk quotient (THQ) level of nitrate (oral and dermal effects) from drinking water exceeds unity for all water samples indicating a potential noncancer risk for the southwestern Nile Delta residents. The potential sources of nitrogen compound pollution are water from sewage treatment plants used for irrigation, sludge and animal manure, septic tanks, soil nitrogen, and artificial fertilizers according to results of δ^15^N values. Results of ammonium/nitrate modeling in shallow groundwater aquifers are compared with observed concentrations and are found to be in good agreement. Some recommendations are given to decrease nitrogen loads in the study area through suggested a need for adoption of N-fertilizer management practices and treatment of sewage water before to application in agricultural activities.

## Introduction

Water is the most important source for the sustenance of humans, especially for drinking industrial and irrigation needs. The necessity for water is increasing significantly for satisfying rapid urbanization rate, overall growth of human population, agricultural intensification, as well as expansion of industry requirements. People rely on surface water, as the principal resource of freshwater, to support their water demands. In many arid and semi-arid areas of developing countries, surface water resources are limited and mostly inadequate because of its contamination, scarcity of precipitation and excessive rates of evaporation exacerbated by climate changes (Khmila et al. 2021). Consequently, reliance on groundwater resources has dramatically increased in these regions (Zhou et al. 2020). However, due to its continuous and instant availability, steady temperature, widespread distribution, limited vulnerability, and natural protection against microbial pollution, groundwater is considered an immensely indispensable resource as a safe reservoir of natural good-quality water, which supports human survival, ecological diversity, and economic development. Widespread groundwater pollution can be caused naturally by geological phenomena such as rock weathering, ore formation, and circulation through different rock and soil types (Bodrud-Doza et al. 2019). Additionally, with the extremely economic development and continued mismanagement, quantity and quality of groundwater resources are adversely affected by numerous causes: global climate changes with scanty rainfall, rapid growth of population and urbanization, intensive increase in agricultural activities with excessive fertilizers application, uncontrolled treated/untreated wastewater discharges, escalated and unplanned industrialization, and mismanaged exploitation of natural resources (Green et al. 2011; Zhang et al. 2015). These intensive human activities may lead to shortage of water resources, increasing deterioration of groundwater quality at an alarming rate by producing various types of pollutants discharged into the water and, subsequently, threatening the integrity of humans, nature, and ecosystems worldwide. Anthropogenic pollution of groundwater is a concealed, intricate, and complicated process with hazardous long-term impacts. Prevention, effective control, and regular monitoring of groundwater pollution should be given priority to protect groundwater resources and to avoid its remediation procedures that can need difficult and expensive techniques (Sidibe and Xueyu 2018). An appropriate strategy for protecting water quality needs to recognize sources of pollution and other factors that control variations in the physical and chemical content of groundwater. Nitrogen (N) is a significant and vital element that organizes the functions and dynamics of ecosystems. It exists in many different forms, including nitrate (NO_3_^−^), nitrite (NO_2_), and ammonium (NH_4_^+^). Nitrate and ammonium are considered to be the most hazardous and ubiquitous nitrogenous pollutants in surface and groundwater that can cause negative impacts on the environment, ecology, and human health. Natural sources of nitrate in aquifers are generally derived from natural fertilization, atmospheric deposition, and bacterial production (Serhal et al. 2009; Ducci et al. 2019; Adimalla et al. 2020; Karunanidhi et al. 2021; Wu et al. 2021), whereas atmospheric nitrogen fixation, mineralization of organic nitrogen, ammonification of dissimilated nitrate by reduction, and re-release from soil under certain conditions are the main natural sources of ammonium in groundwater. However, the excessive nitrogenous contaminants (e.g., nitrate and ammonium) in surface and groundwater systems may be originated from various anthropogenic sources including animal manure, untreated industrial and sewage wastewater disposal, intensive use of pesticides and synthetic N-fertilizers combined with flood irrigation, soil organic nitrogen as well as wastewater treatment plant effluents, which have greatly aggravated surface and groundwater pollution worldwide (Almasri and Kaluarachchi 2007; Su et al. 2013; Chen et al. 2016; Zhai et al. 2017a, b). Excessively high levels of nitrogenous compounds in groundwater, when consumed as drinking water, can cause numerous human health impacts such as infant methemoglobinemia (blue baby syndrome), thyroid enlargement, birth defects, and increase the incidence rates of different cancer types in adults. Furthermore, elevated nitrate concentrations in surface water results in acidification, eutrophication and algal blooms (Bastani and Harter 2019) and it is considered as one of the important threats to aquatic ecosystems. As a result, both the World Health Organization (WHO) and Environmental Protection Agency (EPA) have established permissible nitrate concentration limits in both groundwater and drinking water of 50 mg-NO_3_/L as maximum contaminant level (MCL) (WHO 2017; US EPA 2000), whereas the European Community has derived a limit of 0.5 mg-NH_3_/L for drinking water (EC 1998), since the normal level of ammonium ion in water does not cause a direct threat to human health. Therefore, accurate elucidation of various N pollution sources and effective determination of its transformation mechanisms in surface and groundwater system are essential to develop optimal and sustainable management for water quality preservation (Kendall et al. 2007). Over the recent decades, pollution of surface and groundwater by ammonium and nitrate from anthropogenic sources is a hot topic for scientific researchers and water management organizations as a serious environmental problem (Zhang et al. 2018; Xin et al. 2019).

Nitrogen isotope compositions have frequently been used to discern sources and transformation mechanisms of nitrogen pollutants in contaminated surface and groundwater systems. Nitrate from various sources has distinctive^15^N/^14^N ratios. For examples, the values of δ^15^N-NO_3_ range from − 13 to + 13‰ in atmospheric N deposition and from − 6 to + 6‰ in urea and synthetic ammonium fertilizers (Flipse and Bonner 1985). Manure and sewage have similar isotopic values range from + 4 to + 25‰ (Xue et al. 2009), whereas δ^15^N-NO_3_ values range from 0 to + 8‰ in soil nitrogen. Unfortunately, the δ^15^N contents of two or more nitrate sources mentioned before can sometimes overlap, so the sources cannot be clearly distinguished. In addition, mixing between different sources and complexity of biogeochemical transformation mechanisms (e.g., nitrification, mineralization, biological fixation, denitrification, and volatilization), that affect the isotopic signature, complicates the identification of the main sources of nitrate in groundwater systems. To avoid these problems, different approaches, such as physico-chemical properties, multiple isotopic analyses, in conjunction with multivariate statistical methods and transport modeling, have gradually utilized as effective tools for tracking the various potential sources and transformation pathways of nitrogen pollutants in surface and groundwater systems (Si Liang 2010; Torres-Martínez et al. 2020; Kruk et al. 2020). To our knowledge, researchers have recently attempted to link dual δ^15^N_NH4_ and δ^15^N_NO3_ values with hydrogeochemical properties of water to recognize sources and potential transformation processes of ammonium or nitrate in water systems (Kaushal et al. 2006; Mitchell et al. 2003; Kendall and Aravena 2000; Wilson et al. 1994; Du et al. 2017). Application of nitrogen isotopes (δ^15^N_NH4_ and δ^15^N_NO3_) to distinguish among different pollution sources and nitrogen transformations in surface and groundwater are reported by many authors in Egypt with particular emphasis on the Nile Delta region (e.g., Aly et al. 1982; Ahmed et al. 2007; Ahmed et al. 2009; Ghoraba 2009; Hussien 2005; Aly et al. 2010; Hussien 2011; Ahmed et al. 2013) and worldwide studies (Choi et al. 2003; Lima et al 2015; Lee et al 2020; Wu et al 2021). The reuse of treated and untreated wastewater for irrigation is expanding worldwide to improve water use and recycle nutrients. Mostly, sewage effluents are applied by flood irrigation that enhances fast infiltration of large volumes of water coupled with nutrients to the groundwater leading to aquifer nitrogen pollution. In this study, southwestern Nile Delta, Egypt, was taken as a pilot area that suffers from surface and groundwater deterioration due to excessive use of treated/untreated sewage effluent for irrigation purposes causing human health hazards. The study area is currently served by sewerage system including integrated wastewater treatment plants (Zenin WWTP). The combined primary and secondary treated wastewater, with agricultural and industrial wastes, are discharged to some drainage channels combined with the untreated excess flow that disposed directly to the nearby main drains (Nahya drain, Al-Mariotya drain, Al Rahawy drain) then to Rosetta branch. Where, Al Rahawy drain receives about 450,000 m^3^/day of secondary treated wastewater from (Zenin WWTP) and about (1,450,000 m^3^/day) of primary treated wastewater from the (Abu-Rawash WWTP) (Elewa et al. 2009). This represents source for surface water pollution with different kinds of pollutants, until reaching the Nile River, the final discharge point of wastewater (El Bourie 2008) with population growth and increasing demand for food, many farmers in the downstream areas are using the sewage effluent as nutrient source for agriculture purposes despite the potential public health risks. Few studies were done in order to study the environmental impact of treated wastewater discharge on groundwater aquifer system (El-Fakharany 2013; Mostafa 2015; Awad and El-Fakharany 2020) confirmed that contaminants migrate downward the aquifer and extending into the unconfined highly vulnerable aquifer part, which affected groundwater sustainable use. The main objectives of the present study are the application of hydrogeochemistry, multiple environmental stable isotopes (δ^2^H-H_2_O, δ^18^O-H_2_O, δ^15^N-NH_4_, and δ^15^N-NO_3_) as well as Geographic Information System (ArcGIS) coupled with nitrogen transport numerical modeling (MODFLOW-MT3D) to explore the apportionment of specific and potential sources and controlling migration and transformation mechanisms of nitrate and ammonium of surface and groundwater in Southwestern Nile Delta, Egypt.

### Site description

**Fig. 1:**
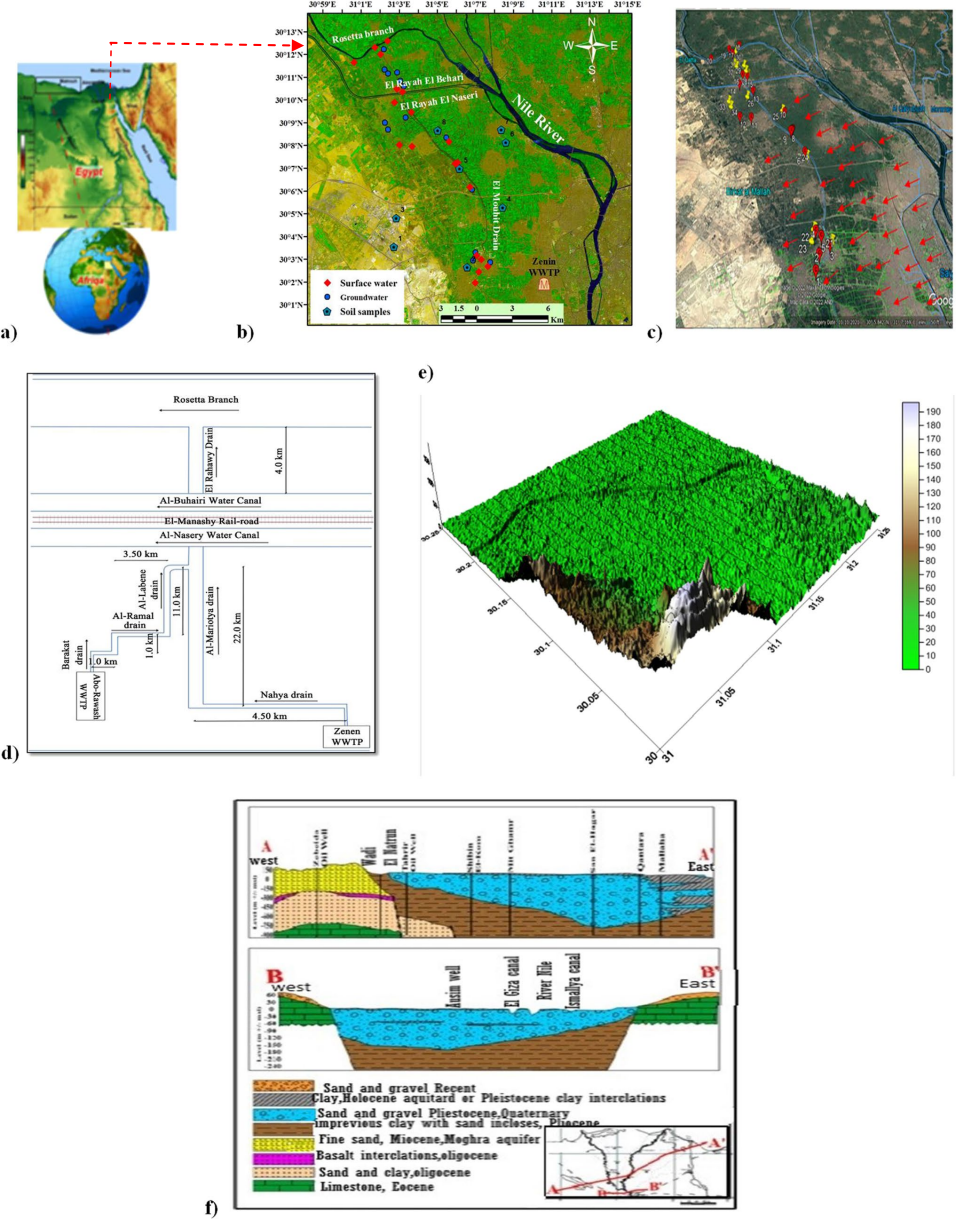
**a** Site map of the study area. **b** Location map of collected surface and groundwater samples. **c** Groundwater flow direction. **d** Sketch diagram for discharging the Abu-Rawash effluent to the Rosetta branch (Mostafa 2015). **e** 3D digital elevation model (DEM). **f** Hydrological cross sections after (RIGW 1989)

The study area is located in southwestern Nile Delta, Egypt, between longitudes from 31° to 31° 10′ Eand latitudes from 30° to 30° 10′ N **(**Fig. 1b). This area suffers from different anthropogenic pollution sources including animal manure application, untreated industrial and sewage wastewater disposal, intensive use of pesticides and synthetic N-fertilizers combined with flood irrigation, soil organic nitrogen as well as wastewater treatment plant effluents that led to water resources quality deterioration which greatly affects surface and groundwater systems in the study area. The study area was dissected by numerous numbers of drains and canals (Nahya drain, Al- Ramal drain, Barakat drain…) that are giving a chance for treated/ untreated wastewater and excess irrigation water to leak down the subsurface causing groundwater aquifer deterioration as illustrated in (Fig. 1b).

### Geology and hydrogeology settings

The regional surface of the area lying to the west of the Nile Delta is covered by sediments and sedimentary rocks ranging in age from the late Cretaceous to Quaternary, Shata (1961). The oldest exposed sedimentary rocks are represented by late cretaceous rocks formed of limestone and chalk. These rocks have a local occurrence on the crest of the complicated folded structure of Abu Rawash area. Mid-Tertiary basalt sheets are the only exposed volcanic rocks in the area. The investigated area comprises surface and subsurface stratigraphic units ranging in age from the Upper Cretaceous to Recent. The surface water system includes the El Mansuriya canal that is passing through pervious and semi-pervious layers. There is direct contact between surface water and subsurface. There are many open drains in the study area that are giving a chance for the wastewater and excess irrigation water to move down to the subsurface causing recharge to the groundwater aquifer. The Quaternary aquifer consists of consecutive layers of sand and gravel intercalated by clay lenses. Its maximum thickness is about 200 m in the northern part and decreases to the south reaching 100 m in the south. Also, the aquifer thickness vanishes westward until the Quaternary sediments overlap with the tertiary sediments of Miocene, Oligocene, or older rocks (Fig. 1e). The thickness of Miocene sediments is about 50 m of sand and clay. Oligocene basalts are occasionally underlying the Miocene and overlying the Oligocene sediments. The layer forming the Quaternary aquifer is saturated with groundwater, especially the northeastern portion. The groundwater exists dominantly under free water table conditions (unconfined) to semi-confined.Where semi-pervious clay cap covers the aquifer, especially in the eastern parts. The air temperature (T) rises in summer with maximum.

Average values exceed 40 °C during July, while minimum average values below 14 °C are measured during January. The relative humidity (RH) reaches its maximum average value of 55% in July and August and its minimum value of 37% in April (Sharaky et al. 2017). Evaporation intensity (Ev) is a significant factor affecting water quality, particularly where groundwater is close to the ground surface. The rainfall effect on the surface and groundwater is insignificant, especially during summer due to limited quantity (UNDP 2013).

## Materials and methods

### Sampling collection and Analytical procedures

A set of representative water samples (20 canals and drainage water) and 14 groundwater samples were collected and analyzed for in situ field measurement physical measurements including electrical conductivity (EC meter, 3510 Jenway-UK), pH, temperature, total dissolved solids (TDS), and dissolved oxygen (DO) using portable probes. Chemical parameters measures comprise major cations (Ca, Mg, Na, K) and anions (Cl, SO_4_, HCO_3_) concentrations. All the wells located in the studied area had been in use during the time of sampling (Fig. 1b). The depths of the selected wells ranged from 11 to 60 m below the surface. The samples were collected in 1-L narrow neck pre-washed polyethylene bottles. Analysis of the water samples was carried out following the methods described in (APHA, Awwa, WPCF 1995). Total hardness (TH) as CaCO_3_ and Ca^2+^ were analyzed using standard EDTA. Mg^2+^ was calculated by taking the differential value between TH and Ca^2+^ concentrations. Na^+^ and K^+^ were measured using a flame photometer. Total alkalinity and CaCO_3_, CO_3_^2−^, and HCO_3_^–^ were estimated by titrating with HCl. Cl^−^ was determined volumetrically by standard Hg (NO_3_)_2_ titration. SO_4_^2−^ and NO_3_^–^ were analyzed using UV/Visible spectrophotometer. Trace element contents for (Pb, Cu, Cd, Co, Ni, and Zn) were determined using atomic absorption spectroscopy (AAS). All parameters are expressed in milligrams per liter and milliequivalents per liter. Data quality was assessed using the charge balance between the difference of cations and anions (expressed in meq/l) divided by their summation according to the following equation:

1 $$\mathrm\Sigma(\mathrm{Cations}-\mathrm{Anions})/\mathrm\Sigma(\mathrm{Cations}+\mathrm{Anions})\times100$$

With an acceptable range of ± 5 (Hem 1991) that confirm the water quality assessment.

### Human health risk assessment

The noncancer health risk associated with drinking nitrate-contaminated water was assessed herein. A method for estimating the total risk quotient (THQ) by the US EPA Regional Risk-Based Concentration was used. Total risk quotient (THQ) is the sum of the oral (HQo) and dermal (HQd) risk quotients. Values of THQ < 1 suggest an acceptable non-carcinogenic risk, while values of THQ > 1 indicate a potential risk to human health (Liu et al. 2019; Wagh et al. 2019). The risk associated with the non-cancer effects of nitrate through drinking water is expressed as follows:

2 $$HQo=\frac{Cw\times EF\times ED\times IR}{RfDo\times BW\times AT}$$

3 $$HQd=\frac{Cw\times Ki\times CF\times EF\times ED\times EV\times SA}{RfDd\times BW\times AT}$$

where Cw = the nitrate concentrations (mg/l), EF = the exposure frequency (350 days/year), ED = the duration of exposure (30 years), IR = the amount of water ingested by an adult, (2L/day), RfD = the oral reference dose (1.6 mg/L/day of NO_3_-N), BW = the body weight of an adult (60 kg), AT = the averaging time for non-carcinogens (30 years × 365 days/year = 10,950 days), EV  Daily exposure frequency of dermal contact, SA= 239 XH^0.417^ X BW^0.517^. An acceptable standard human health risk by drinking water is a THQ value of under unity.

### Stable isotope analysis

Environmental stable isotopes (δ^18^O and δD in ‰) were measured according to the procedure described in Clark and Fritz (1997). Both results were expressed in δ‰ with the analytical errors of ± 0.1 and ± 1‰ for δ^18^O and δD, respectively. The stable isotope data are reported vs. Vienna-Standard Mean Ocean Water (V-SMOW) in ‰ using δ notation according to the equation:

4 $$\Delta = [({\mathrm{R}}_{\mathrm{sample}}/{\mathrm{R}}_{\mathrm{standard}})-1] \times 1000$$

R _sample_ and R _standard_ are the measured isotopic ratios (^18^O/^16^O) and (^2^H/^1^H) of sample and standard material.

For nitrogen isotope analysis, samples were collected in polyethylene bottles and acidified using 1 ml concentrated.H_2_SO_4_ per 1L water sample to prevent bacterial growth that enhances ammonia/nitrate degradation. The vacuum distillation method was used for the determination of ammonium and nitrate in water samples (Aly and Mohamed 2008). Soil samples were collected and air dried at room temperature and then ground to pass a 2-mm screen. The ammonium and nitrate in soil were extracted by the three-fold amount of 2 M KCl. The extract was filtered using filter paper (Whatman No. 42) then the separation of ammonium and nitrate in soil extracts was done using (Vacuum Distillation Method). δ^15^N of dissolved nitrate was analyzed following the oxidation of ammonium sulfate under vacuum with LiOBr and using AIR as reference (IAEA 1995). The analytical error was ± 0.5%. All gases were analyzed on a Finnigan Thermo Quest Delta plus XL mass spectrometry. Ground surface elevation and the location of the sampling sites were recorded using a Global Positioning System (GPS) manufactured by GARMIN. This was supported by a topographic sheet made available by the Egyptian Survey Department. Measured and estimated groundwater variables were analyzed within ArcGIS 10.2.2 package. All the chemical and Environmental isotope analyses were carried out in the Central Laboratory of Environmental Isotope Hydrology at Egyptian Atomic Energy Authority (EAEA).

### USGS-MODFLOW 2000 computer code

The USGS-MODFLOW computer code was used to build a three dimensional model of subsurface water flow. The model describes ground water flow under non-equilibrium conditions in a heterogeneous and anisotropic medium according to Eq. (1) (Bear 1979; Bear and Verruijt 1987).The model is capable of simulating the time-dependent flow as well as mass and heat transport. The time-dependent data which are included in the Finite Difference Model (FDM) has to be stored outside into a database or GIS systems. The governing equations are derived through combination between the water balance equation and Darcy’s law (Anderson and Woessner 1992). The model describes groundwater flow of constant density under non-equilibrium conditions in a heterogeneous and anisotropic medium according to the following equation which was solved using the finite difference technique:

5 $$\frac{\partial }{\partial x}\left({K}_{xx}\frac{\partial h}{\partial x}\right)+\frac{\partial }{\partial y}\left({K}_{yy}\frac{\partial h}{\partial y}\right)+\frac{\partial }{\partial z}\left({K}_{zz}\frac{\partial h}{\partial z}\right)-W={S}_{s}\frac{\partial h}{\partial t}$$

where *K*_*xx*_, *K*_*yy*_, and *K*_*zz*_ are the hydraulic conductivity along the *x*, *y*, and *z* coordinate axes, (Lt-l); *h* is the potentiometric head (L); *W* is a volumetric flux per unit volume and represents sources and/or sinks of water (t); *S*_*s*_, is the specific storage of the porous material (L-l); and *t* is time (t). In general, *S*_*s*_, *K*_*xx*_, *K*_*yy*_, and *K*_*zz*_ may be functions of space (*S*_*s*_ = *S*_*s*_(*x*, *y*, *z*), *K*_*xx*_ = *K*_*xx*_(*x*, *y*, *z*), etc.) and W may be a function of space and time (*W* = *W*(*x*, *y*, *z*, *t*)). Moreover, due to the hydrodynamic dispersion, the concentration of a solute will decrease over distance. Generally speaking, the solute will spread more in the direction of groundwater flow than in the direction normal to the groundwater flow, because longitudinal dispersivity is typically 10 times higher than transverse dispersivities. The transport of a conservative solute in a one-dimensional system can be described by the advection–dispersion Eq. (2):

6 $$\frac{\partial C}{\partial t}=-v\frac{\partial C}{\partial x}+D\frac{\partial 2C}{\partial x2}$$

where ∂C/∂t is the change in concentration over time, the first term on the right-hand side represents advection and the second term represents hydrodynamic dispersion. The advection–dispersion equation may be solved analytically or numerically under different initial and boundary conditions.MT3DMS was used to predict heavy metal transport using lead as the surrogate. This engine is the best choice when biodegradation is not a factor when dealing with heavy metals that are persistent (Prommer et al. 2002). The adsorption coefficients were also most appropriate with the MT3DMS engine with heavy metal only having one oxidation state. The model was run for numerous iterations and outputs were recorded at different time intervals to show the size and extent of the heavy metal plume.

#### Spatial and temporal model discretization

**Fig. 2:**
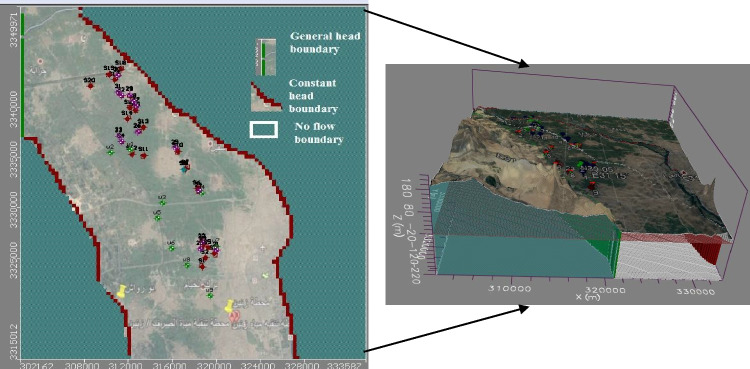
Finite-difference grid and boundary conditions for southwestern Nile Delta shallow aquifer

The model domain and finite difference grid used to simulate groundwater flow in the southwestern Nile Delta shallow aquifer are illustrated in (Fig. 2). The model encompasses an area of about 0.11317 km^2^. The grid consists of 100 rows and 100 columns with 10,000 regular cells in plain view. Each cell is 35.76 × 31.647 m^2^ in the horizontal plane. Two layers have been modeled: (1) clay cap layer covering the aquifer with a thickness of 15 m to the east and vanishing westward (Al-Agha et al. 2015), (2) Quaternary aquifer layer with a maximum depth of 200 m. Boundary conditions were selected depending on the hydrogeological and geological conditions of the studied area. The eastern and western boundary is a constant head boundary coinciding with El Rayah El Behari and El Mansouria canal with a piezometric head line of + 14 and + 12 m (amsl), respectively. The southwestern boundary is no flow boundary. The northwestern part was a general head boundary with a piezometric head line + 11 m. The horizontal hydraulic conductivity of the groundwater aquifer ranges from 50 to 75 m/day and decreases to 40 m/day in the phreatic aquifer to the west of the study area. The horizontal permeability of the semi- pervious clay cap layer ranges from 1.0 to 2.0 m/day, while the vertical permeability ranges from 0.01 to 0.2 m/day (RIGW 2002). The nitrate transport process was modeled in MT3D which employs a mixed Eulerian–Lagrangian approach to solve the advection–dispersion reaction equation (Zheng 1990).

#### Calibration flow and transport model in steady state

**Fig. 3:**
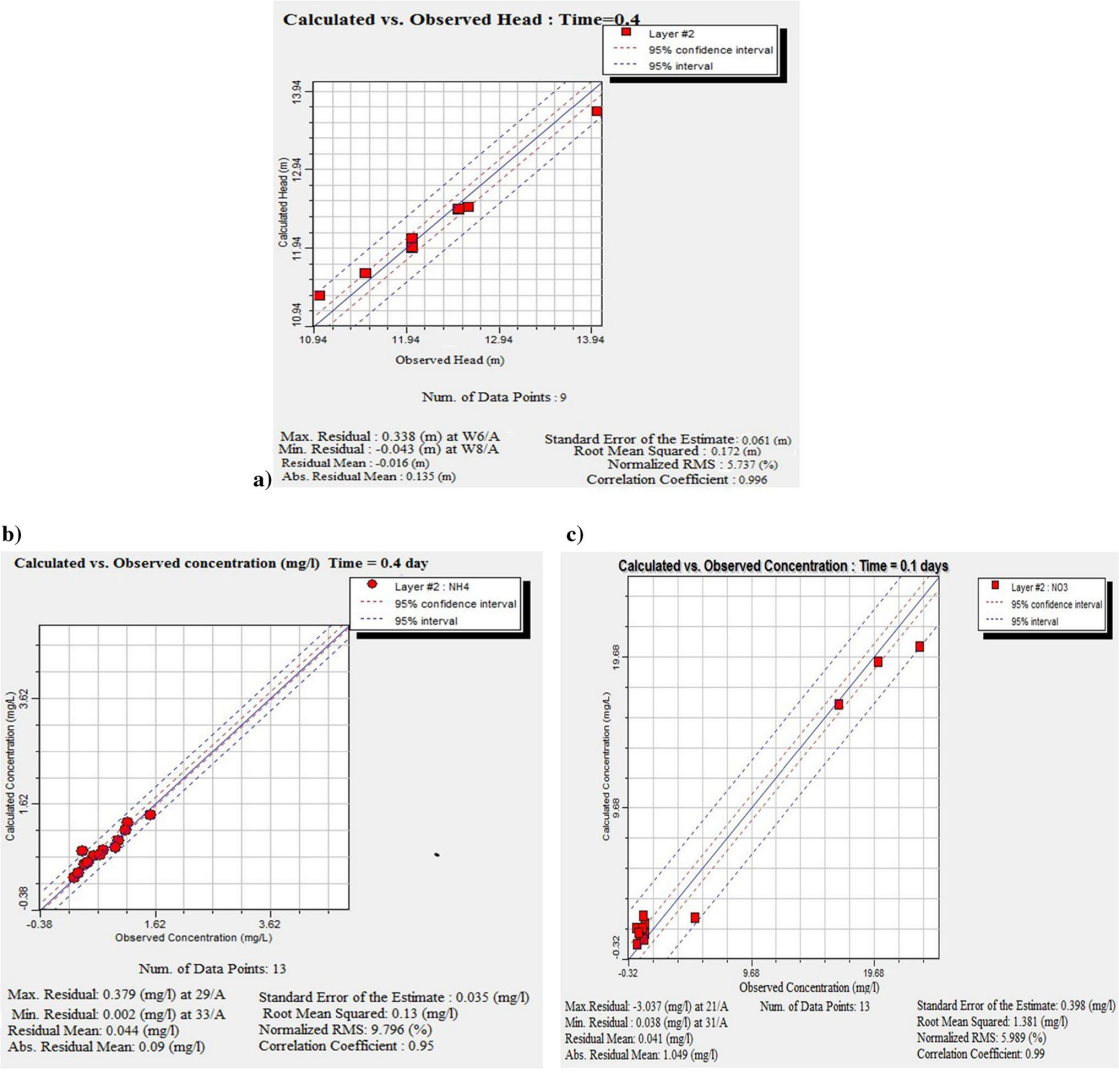
**a** Calibration of observed and calculated heads in 2004. **b** Observed vs. calculated calibration of NH_4_^+^ concentration; **c** Observed vs. calculated calibration of NO_3_^−^ concentration

Model calibration was achieved through trial and error by adjusting the values of recharge at the boundaries, hydraulic conductivity, and initial head. The calibration has been conducted versus potentiometric head data in 2004 to calibrate the spatially variable hydraulic conductivity and recharge, and hydraulic conductivity of the general head boundary. The calibration process produced an acceptable comparison between observed vs. calibrated heads and concentration in (mg/l) (Fig. 3a–c).

## Results and discussion

### Surface and groundwater geochemistry

Summary of descriptive statistical results (minimum, maximum, mean, and standard deviation) of physical and chemical analysis of collected surface and groundwater samples are illustrated in Table 1. Values of pH in surface water varied from 6.82 to 8.82 with mean of 7.96 indicating a slightly acidic to alkaline character. While for groundwater samples varied from 7.27 to 8.64 with mean of 8.08 suggesting neutral to alkaline character. It within safe limit of 6.5–8.5 for drinking purpose according to WHO (2017). The TDS is ranged between 256 to 2451 mg/l with mean value of 809 mg/l. In 15% of surface water samples, TDS values exceeding WHO limits of 1000 mg/l. While, TDS values ranged from 530 to 2410 mg/l. In 71% of groundwater samples exceeding the safe limit of drinking which may causes gastrointestinal irritation (WHO 2017). As dissolved oxygen (DO) values refer to the level of free oxygen present in water; it is a vital parameter in water quality indicators. Where, lower values than the permissible standards may be indication of anthropogenic pollution problems. DO in surface water samples ranged from 0 to 10 mg/l with mean value of 2.02 mg/l. For groundwater samples, it ranged from 0.62 to 2.35 mg/l with mean value of 1.46 mg/l. Among the major cations and anions, Na^+^ (20.76–319 mg/l) and HCO_3_^−^ (122–626 mg/l) dominate surface water samples followed by Ca^2+^ (28–195 mg/l), Mg^2+^ (10.96–93.5 mg/l), K^+^ (1.83–79 mg/l), and SO_4_^2−^ (42.8–544.5 mg/l) and Cl^−^ (11.3–430.7 mg/l), respectively. While, for groundwater samples, the major cations and anions dominance was Ca^2+^ (40.88–153 mg/l) and HCO_3_^−^ (180–617.9 mg/l) followed by Na^+^ (27–158.5 mg/l), Mg^2+^ (13.94 to 117.6 mg/l), K^+^ (3.8 to 27.3 mg/l), Cl^−^ (28.9 to 405 mg/l), and SO_4_^2−^ (10.6 to 264 mg/l), respectively. According to the ion concentrations of the surface and groundwater samples in the studied area, the hypothetical salt combinations are classified into four groups:

- *Group 1*: NaCl, Na_2_SO_4_, MgSO_4_, Mg(HCO_3_)_2_, Ca(HCO_3_)_2_ (low-salinity meteoric water) including about 75% of surface water samples (sample nos. 2, 6, 7, 8, 9, 10, 11, 12, 13, 14, 15, 16, 17, 18, and 19).
- *Group 2*: NaCl, Na_2_SO_4_, MgSO_4_, CaSO_4_, Ca (HCO_3_)_2_ (mixed water) including about 57% of groundwater samples (sample nos. 22, 24, 25, 26, 27, 28, 33, and 34) and about 10% of surface water samples (sample nos. 1 and 4).
- *Group 3*: NaCl, Na_2_SO_4_, NaHCO_3_, Mg (HCO_3_)_2_, Ca (HCO_3_)_2_ (meteoric water) including about 14% of groundwater samples (sample nos. 21 and 30) and about 10% of surface water samples (sample nos. 3 and 20).
- *Group 4*: NaCl, MgCl_2_, MgSO_4_, CaSO_4_, Ca (HCO_3_)_2_ including about 28% of groundwater samples (sample nos. 23, 29, 31, and 32).

**Table 1:** Descriptive statistical summary of physical, chemical, saturation index, and trace elements results for water samples

Sample		Min	Max	Mean	SD	Sample	Min	Max	Mean	SD
Surface water	pH	6.82	8.82	7.96	0.54	Groundwater	7.27	8.64	8.08	0.38
	Depth (m)						11	60	26.79	14.44
	EC (µS/cm)	400	3830	1264.30	773.51		530	2410	1342.43	491.56
	TDS (mg/l)	256	2451.2	809.15	495.05		339.2	1542.4	859.15	314.60
	DO (mg/l)	0	10.45	2.02	2.92		0.62	2.35	1.47	0.56
	Temp (°C)	24.8	31.6	28.74	1.57		22.6	28	24.79	1.53
	Na (mg/l)	20.76	319	111.21	68.58		27.12	158.5	88.14	37.19
	K (mg/l)	1.83	79	16.93	16.96		3.8	27.3	9.86	6.61
	Mg (mg/l)	10.96	93.5	30.09	18.90		13.94	117.6	46.77	26.24
	Ca (mg/l)	28.28	195	60.88	34.55		40.88	153.1	76.23	35.93
	Cl (mg/l)	11.3	430.7	125.58	101.43		28.9	405	127.00	91.91
	HCO_3_ (mg/l)	122	626	258.87	120.95		180.3	617.9	324.02	127.15
	SO_4_ (mg/l)	42.8	544.5	157.24	104.90		10.6	264	144.55	76.58
	NH_4_ (mg/l)	0.29	124.2	19.00	27.91		0.21	1.75	0.79	0.47
	NO_3_ (mg/l)	0.52	39.67	5.58	8.76		0.33	32.8	7.48	10.94
	SI_Calcite_	− 0.484	1.629	0.52	0.53		− 0.164	1.358	0.82	0.44
	SI_Dolomite_	0.098	4.268	2.02	1.11		0.641	3.629	2.73	0.96
	SI_Anhydrite_	− 2.548	− 1.142	− 1.95	0.29		− 3.018	− 1.492	− 1.96	0.42
	SI_Gypsum_	− 2.258	− 0.853	− 1.66	0.29		− 2.728	− 1.202	− 1.67	0.42
	SI_Halite_	− 8.197	− 5.541	− 6.65	0.56		− 7.68	− 5.937	− 6.70	0.45
	THQ	0.918	70.05				0.582	57.92		
	Pb (mg/l)	0.134	2.125	0.646	0.654		0.139	0.649	0.327	0.162
	Cu (mg/l)	0.0	0.272	0.047	0.078		0.002	0.05	0.023	0.013
	Cd (mg/l)	0.455	6.729	2.051	1.862		0.172	7.808	2.256	2.611
	Co (mg/l)	0.0	0.036	0.004	0.008		0.0	0.04	0.008	0.012
	Ni (mg/l)	0.001	0.134	0.027	0.039		0.001	0.04	0.011	0.011
	Zn (mg/l)	0.047	0.559	0.173	0.141		0.008	0.683	0.214	0.217

**Fig. 4:**
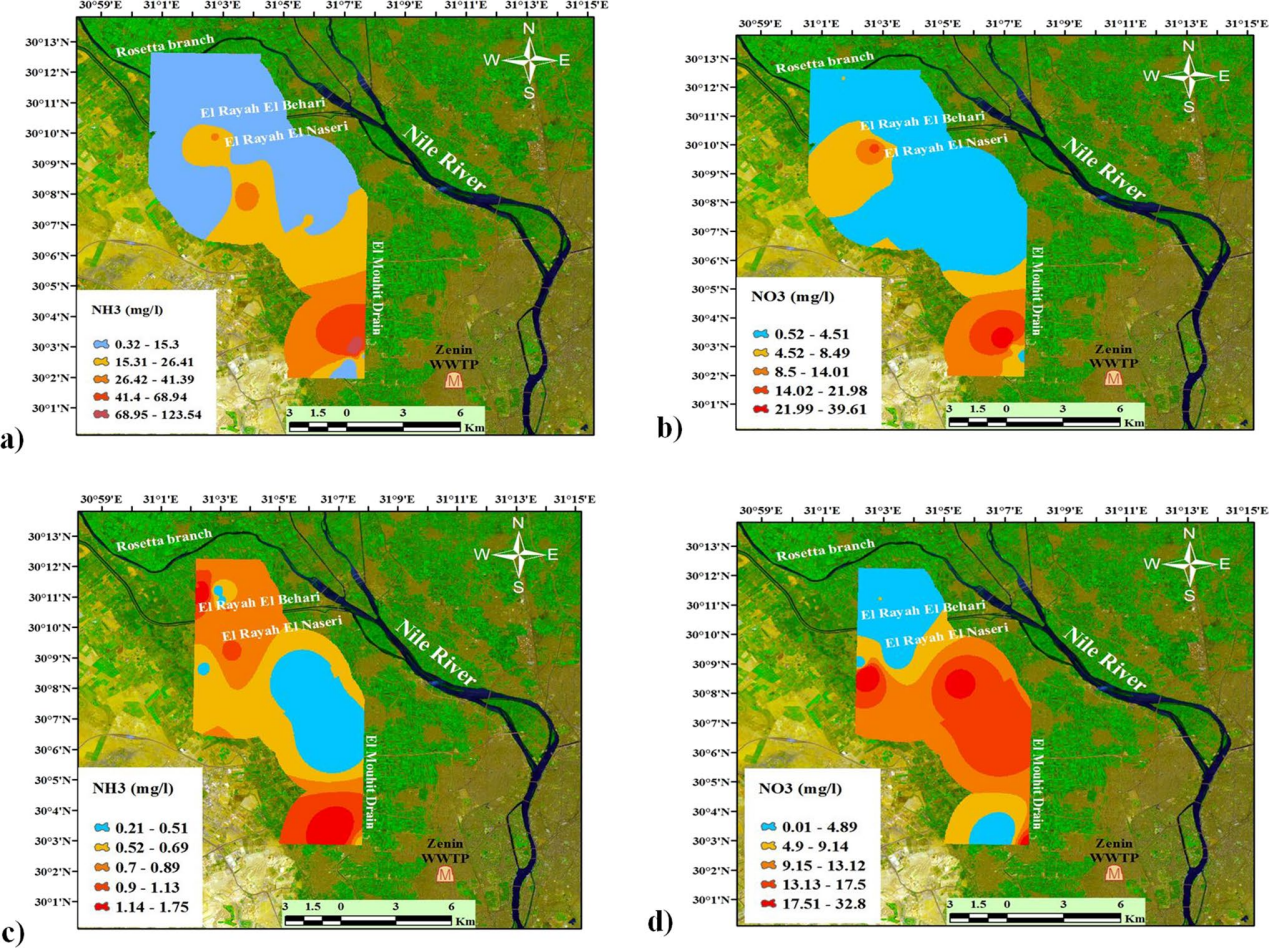
Spatial distribution of NH_3_ and NO_3_ in surface water (**a**, **b**) and groundwater (**c**, **d**)

The spatial distribution of ammonia NH_4_^+^ and nitrate NO_3_^-^ through the study area was illustrated in (Fig. 4). Higher concentrations were observed in the southern and southeastern directions where Zenin Wastewater Treatment Plant (WWTP) was located. Surface water concentration of ammonia NH_4_^+^ and nitrate NO_3_^−^ ranged from 0.29 to 124 mg/l and 0.52 to 39.67 mg/l, respectively. For groundwater samples, NH_4_^+^ and NO_3_^−^ concentrations ranged from 0.21 to 1.75 mg/l and 0.33 to 32.8 mg/l, respectively. The NO_3_^−^ content was above the threshold value for anthropogenic influence 3 mg/l in 40% and 36% and above the drinking water standard (10 mg/l) in 10% and 29% of surface and groundwater samples, respectively.

The saturation index is a vital geochemical parameter in the ields of hydrogeology and geochemistry, often useful for identifying the existence of some common minerals in the groundwater system (Deutsch 1997).When the groundwater is saturated with some minerals, SI equals zero; positive values of SI represent oversaturation, and negative values show undersaturation (Appelo and Postma 1994; Drever 1997). Saturation index results, as illustrated in Table 1, indicated that the surface water (canals, drains) samples were undersaturated (dissolution is required to achieve equilibrium) with minerals and gases in order of cerussite PbCO_3_> gypsum CaSO_4_·2H_2_O> smithsonite ZnCO_3_>anhydrite CaSO_4_> halite NaCl, while groundwater Quaternary aquifer samples have a tendency dissolve the minerals/gases in order of cerussite PbCO_3_> smithsonite ZnCO_3_> gypsum CaSO_4_·2H_2_O> >anhydrite CaSO_4_> halite NaCl and supersaturated with calcite CaCO_3_dolomite CaMg (CO_3_)_2_ for all collected water samples where dissolution-precipitation reactions can be expressed as:

7 $${\mathrm{CaCO}}_{3}+ {\mathrm{CO}}_{2}+ {\mathrm{H}}_{2}\mathrm{O}\leftrightarrow {\mathrm{Ca}}^{2+}+2{\mathrm{HCO}}_{{3}^{-}}$$

8 $$\mathrm{CaMg }{\left({\mathrm{CO}}_{3}\right)}_{2}+ 2{\mathrm{CO}}_{2}+ {\mathrm{H}}_{2}\mathrm{O}\leftrightarrow {\mathrm{Ca}}^{2+}+{\mathrm{Mg}}^{2+}+4{\mathrm{HCO}}_{{3}^{-}}$$

**Fig. 5:**
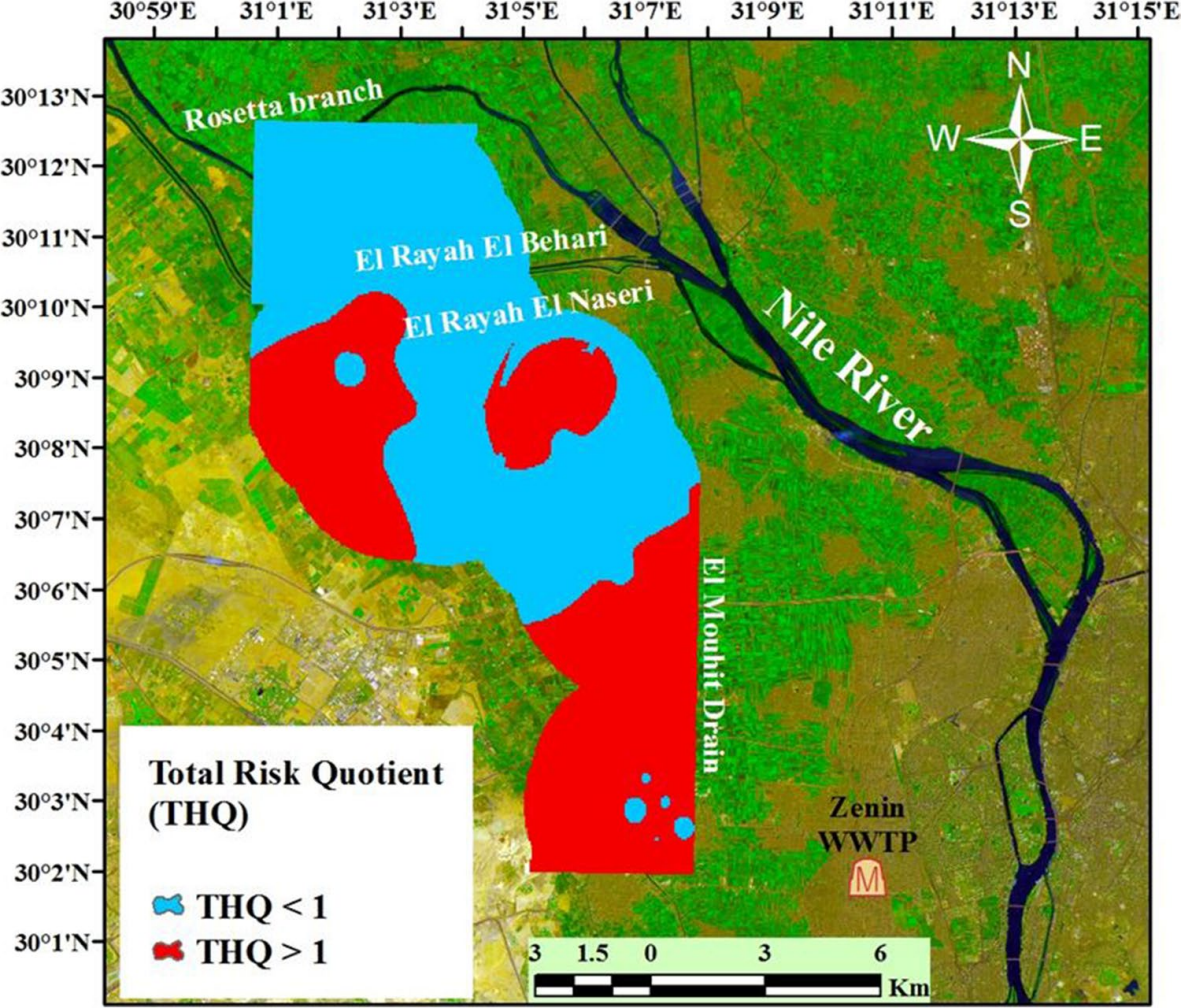
Nitrate total risk quotient (THQ) for collected water samples

Total risk quotient (THQ) level of nitrate (Oral and Dermal effects) from drinking water ranged from 2.36 to 180.1 and 1.5 to 148.9 for surface and groundwater, respectively, taking into consideration that about 5% and 22% of surface and groundwater only are < 1 and the rest of samples are exceeding unity respectively. Spatial distribution of THQ for nitrate is illustrated in Fig. 5, where values of THQ exceed unity was located at western southern and southern parts of the studied area. These locations exhibit high nitrate concentration values and highly population areas where most of their inhabitants depend on groundwater for consumption and domestic activities. Higher values of THQ that exceed unity reveal an acute non-carcinogenic health risk for residents in these areas.

**Fig. 6:**
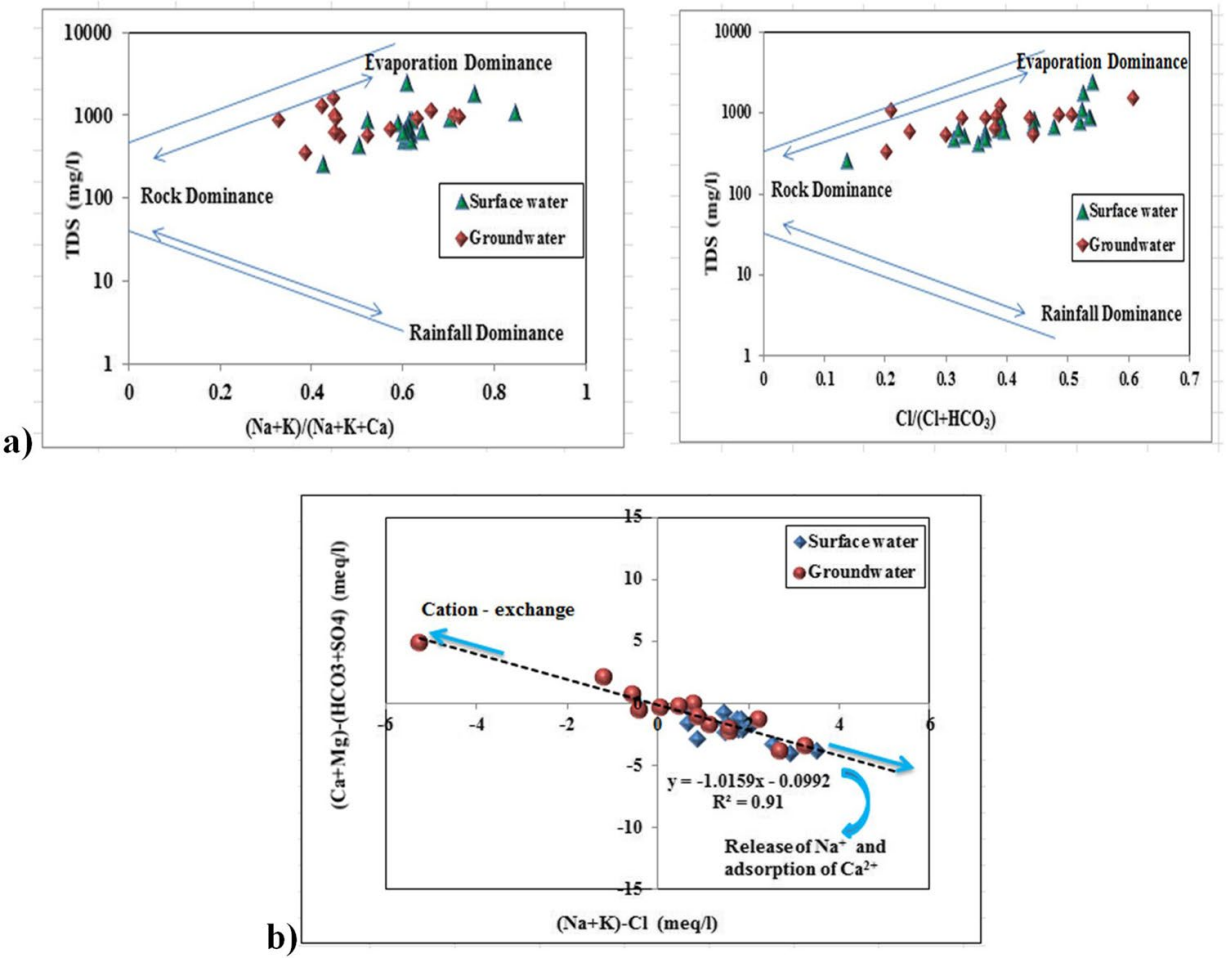
**a** Gibbs plot for the collected water samples. **b** Plot showing the relationship between (Na + K)- Cl vs. (Ca + Mg)-(SO_4_ + HCO_3_)

Gibbs plot introduced a presentation that helps to differentiate between the samples according to their chemical compositions which could be affected either by rock weathering or evaporation/Rainfall dominance. This classification is based on the relation of TDS vs. (Na/(Na + K) or (Cl/(Cl + HCO_3_) in the cationic and anionic sides respectively. The distribution of the studied surface and groundwater samples on Gibbs diagrams (Fig. 6a) shows that all samples were affected by evaporation process and rock–dominance processes. Hence, a direct cation–exchange reaction had controlled surface and groundwater mineralization, as shown in Fig. 6b, according to the following process:

9 $${\mathrm{Na}}_{2}\mathrm{X}+{\mathrm{Ca}}^{2+} =\mathrm{ CaX }+ 2{\mathrm{Na}}^{+}$$

10 $${\mathrm{Na}}_{2}\mathrm{X}+{\mathrm{Mg}2}^{+} =\mathrm{ MgX }+ 2{\mathrm{Na}}^{+}$$

where X = aquifer solid, Ca^2+^, Na^+^, and Mg^2+^ are calcium, sodium, and magnesium ions, respectively.

### Trace element pollution indicators

**Fig. 7:**
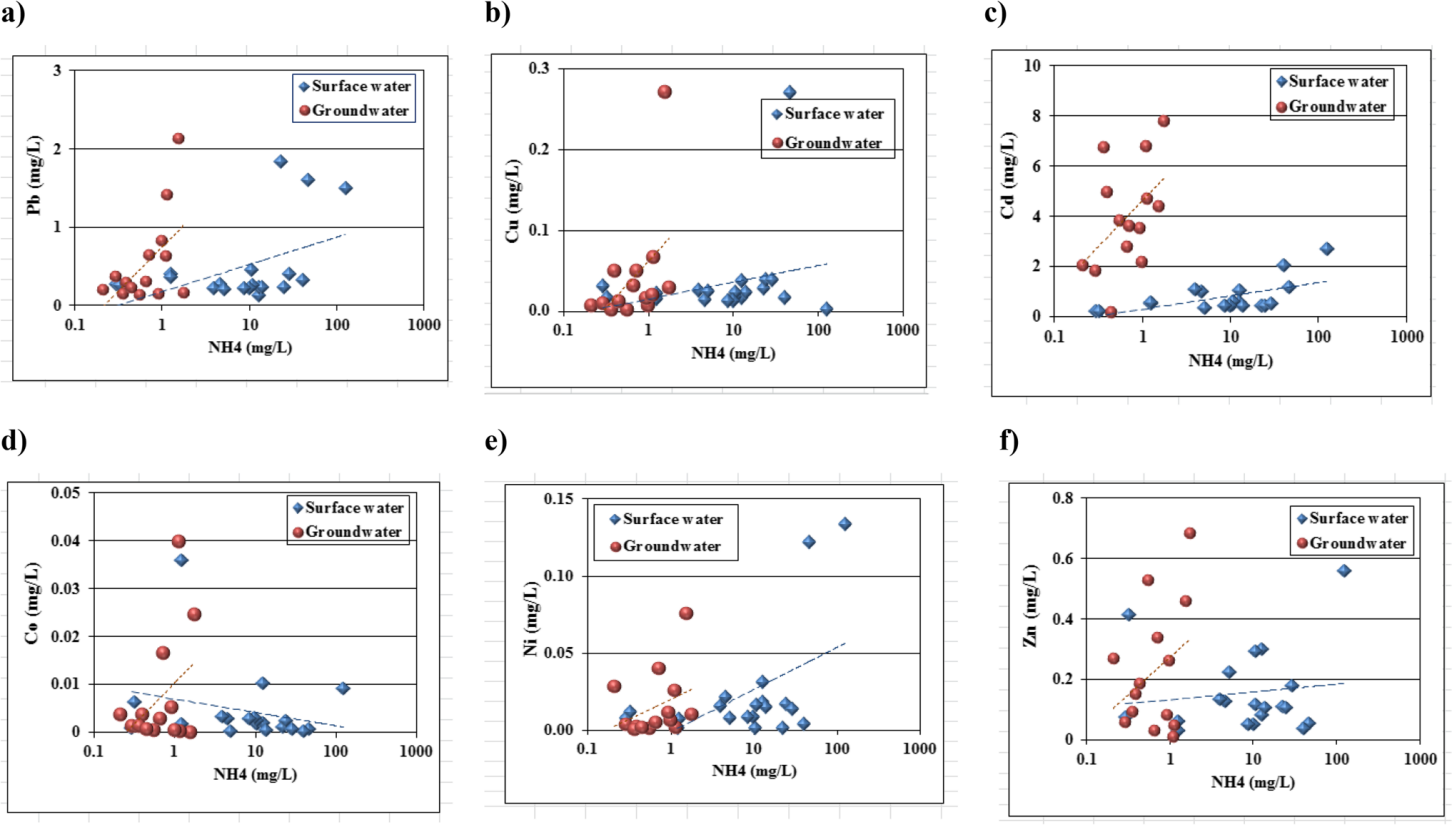
Binary plots of NH_4_ vs. trace elements: **a**) Pb, **b**) Cu, **c**) Cd, **d**) Co, **e**) Ni, **f**) Zn

**Fig. 8:**
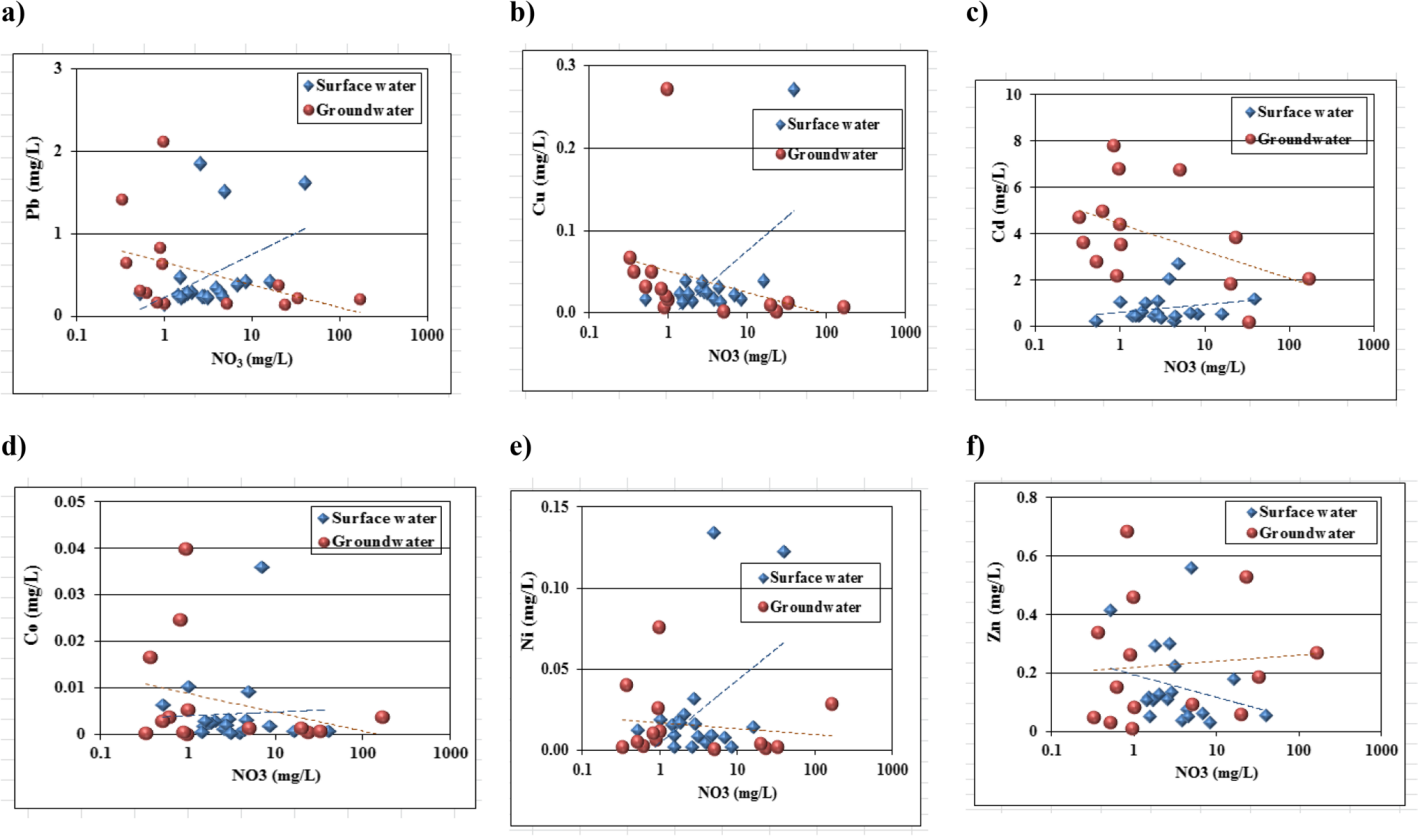
Binary plots of NO_3_ vs. trace elements: **a**) Pb, **b**) Cu, **c**) Cd, **d**) Co, **e**) Ni, **f**) Zn

Statistical summary of trace elements results of surface and groundwater samples illustrated in Table 1. Higher concentration values of Cd, Pb, and Ni compared with maximum concentration levels of trace elements for drinking water (WHO 2017) and (US EPA 2000). Lead concentration ranges from 0.134 to 2.125 mg/l and 0.139 to 0.649 mg/l in surface and groundwater, respectively. Nickel concentration varies from 0.001 to 0.134 mg/l and 0.001 to 0.04 mg/l for surface and groundwater, respectively. It was seen that, the mean concentrations of trace elements followed a descending order as; Cd > Pb > Zn > Cu > Ni > Co for surface water and groundwater samples. Binary plots of trace elements (Pb, Cu, Cd, Co, Ni and Zn) vs. NH_4_ and NO_3_ are shown in (Figs. 7 and 8). As trace elements pollutant sources resulting from human activities include industrial wastewater, agricultural drainage water, sewage water discharge that cause many diseases all over the world. In addition to, application of organic/ inorganic fertilizers may cause soil compaction due to changes in land use and fast urbanization as well as under permanent grassland may reduce the thickness of the aerated zone and lead to anaerobic conditions at subsurface levels in the soil profile. Anaerobic bacteria in these zones may, therefore, reduce the nitrate in the infiltrating water to gaseous nitrogen, causing a decrease in nitrate levels in groundwater in such locations.

### Environmental stable isotopes

Stable isotopes (δ^18^O and δD and δ^15^N) of collected water samples were measured to provide basic information on the origin, sources, pollution sources, migration pathways and mixing between groundwater of the different hydro-stratigraphic units in southwestern Nile Delta area.

#### Isotopic content of δ^18^O and δD

**Fig. 9:**
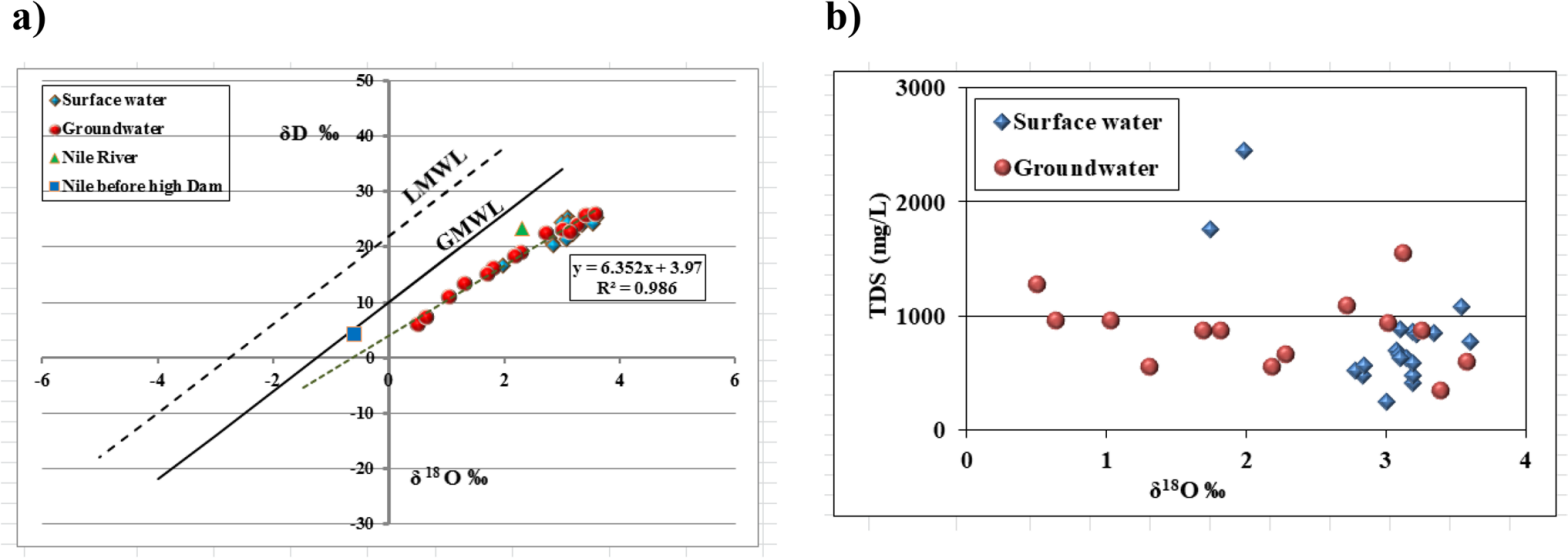
**a** δ^18^O vs. δD relationship in permille (‰). **b** δ^18^O vs. TDS in mg/l relationship

Stable isotope composition of δ^18^O and δD for surface and groundwater samples are summarized in Table 2. Surface water samples show δ^18^O values ranging from 1.74 to 3.6‰ with a mean value of 3.01‰ and δD values range from 15.35 to 25.39‰ with a mean value of 22.67‰. For groundwater samples, δ^18^O values changed from 0.51 to 3.58‰ with a mean value of 2.19‰ and δD values range from 5.96 to 25.96‰ with a mean value of 17.87‰. The relationship between δ^18^O and δD for surface and groundwater samples is shown in Fig. 9a offering a complementary insight into water resources origin of the southwestern area of Nile Delta. Surface and groundwater samples are clustered around a regression line according to their isotopic composition regarding GMWL having an equation of δD = 6.35δ^18^O + 3.98 of slope 6.35 and intercept of 3.98 with a correlation coefficient of *R*^2^ = 0.98. All surface water and some groundwater samples have enriched values for δ^18^O and δD suggesting evaporation process dominance before aquifer recharge. From this dataset, it is noticed that surface water samples are more evaporated than groundwater; this is essential because the irrigation water flooded in the cultivated area undergoes excessive evaporation due to their surface long exposure to the atmosphere where flood irrigation scheme was intensively applied in the study area. This circulation of irrigation water can alter the isotopic composition of canals and drain water, and subsequently, it will be enriched in stable isotopes (δ^18^O and δD). The δ^18^O- TDS relationship suggests variability of δ^18^O values compared with constant values of TDS in mg/l enhancing dissolution process effects.

**Table 2:** Results of environmental stable isotopes of (O, D, and N) for collected water and soil samples

Sample type	Sample no	Depth (m)	δ^18^O (‰)	δ^2^H (‰)	Deuterium excess (‰)	NH_4_^+^ (mg/l)	δ^15^N-NH_4_^+^ (‰)	NO_3_^−^ (mg/l)	δ^15^N-NO_3_^−^ (‰)
Surface water	1		3.6	25.39	− 3.41	1.22	6.93	8.43	10.04
	2		3.34	24.09	− 2.63	1.23	7.01	6.85	11.02
	3		2.83	20.71	− 1.93	12.58	6.78	1.01	
	4		1.98	16.53	0.69	46.38	7.03	39.67	8.1
	5		1.74	15.35	1.43	124.2	4.62	4.91	
	6		2.78	21.67	− 0.57	22.3	3.96	2.56	
	7		3.19	22.27	− 3.25	4.56	12.13	2.05	
	8		3.1	25.31	0.51	10.42	5.19	1.52	
	9		2.84	20.21	− 2.51	24.18	3.51	1.69	
	10		3.54	24.33	− 3.99	3.89	13.01	2.84	
	11		3.22	23.59	− 2.17	39.93	3.8	3.86	
	12		3.19	24.33	− 1.19	0.29		4.39	5.5
	13		3.07	21.39	− 3.17	12.52	5.12	2.74	
	14		3.1	24.38	− 0.42	28.41	4.14	16.12	5.09
	15		3.09	22.82	− 1.90	13.72	4.9	1.43	
	16		3.19	24.44	− 1.08	5.02	4.95	3.09	
	17		3.15	23.79	− 1.41	10.57	5.63	1.83	
	18		3	24.45	0.45	0.32		0.52	
	19		3.19	23.9	− 1.62	9.77	5.83	4.59	
	20		3.1	24.5	− 0.30	8.47	4.49	1.55	
Groundwater	21	19	2.73	22.51	0.67	0.55	0.33	23.39	5.94
	22	22	0.51	5.96	1.88	1.54	1.02	0.98	
	23	42	0.64	7.2	2.08	1.14	2.73	0.33	
	24	11	3.26	24.07	− 2.01	0.21		167.9	4.15
	25	18	1.04	10.88	2.56	0.29		19.97	27.91
	26	22	1.31	13.37	2.89	0.98		0.9	
	27	60	2.29	18.91	0.59	0.92	0.766	1.01	
	28	30	3.02	23.22	− 0.94	0.39	− 0.529	0.62	
	29	16	3.13	22.8	− 2.24	0.36		5.09	
	30	18	3.4	25.78	− 1.42	0.71	− 5.7	0.37	
	31	50	1.82	16.11	1.55	1.11	1.8	0.95	
	32	18	1.7	15.16	1.56	1.75		0.83	
	33	32	2.19	18.29	0.77	0.66	10.8	0.52	
	34	17	3.58	25.96	− 2.68	0.44		32.8	3.93
Soil samples	1					17.1	− 1.41	109.2	5.9
	2					5.12	3.94	1.34	
	3					13.76	− 8.99	15.62	5.75
	4					9.36		41.3	6.23
	5					12.53	− 9.56	2.66	
	6					13.11	− 5.36	6.65	12.85
	7					10.87	− 3.51	33.51	9.76
	8					20.51	− 1.92	3.75

#### Nitrate pollution sources using chemical indicators

For ammonia and nitrate production/consumption there are many factors controlling these processes (size of substrate pool, availability of electron donors, dissolved oxygen concentration, and pH range) (Nikolenko et al. 2018; Rivett et al. 2008). Nitrification of NH_4_^+^ remineralized from the soil is a nitrate production process, generally involving microorganisms, which is favored under conditions of DO > 4 mg/l and pH values ranging between 6.5 and 8.

**Fig. 10:**
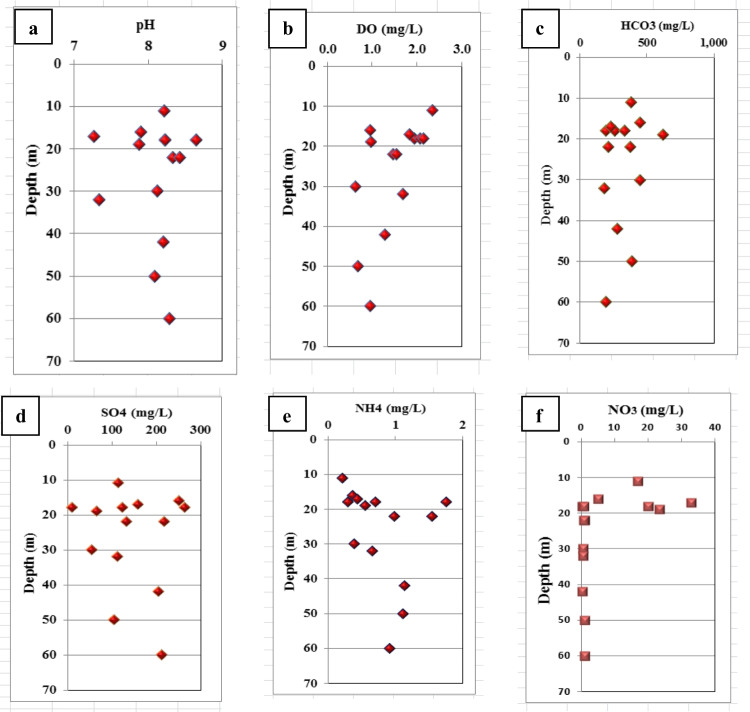
Physical and chemical parameters vs. groundwater depth in (m)

**Fig. 11:**
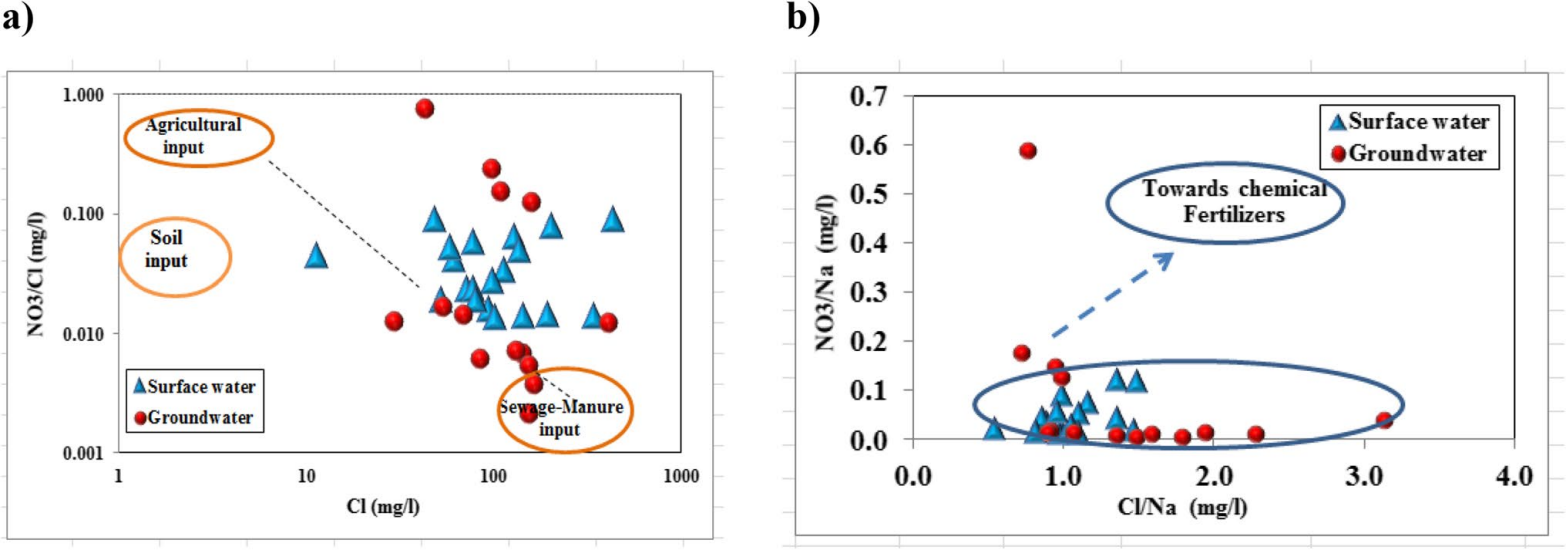
Binary plots of **a** NO_3_/Cl vs. Cl. **b** Cl/Na vs. NO_3_/Na relationship for collected water samples

The results of this process are an increase in NO_3_^−^ concentration and depletion in the δ^15^N isotopic composition (Kendall 1998). Ammonium and nitrate concentrations in groundwater samples were illustrated in Table 2. For collected water samples in the study area, different physical and chemical parameters affect ammonium and nitrate distribution with depth in (m) as shown in Fig. 10. As groundwater depths ranged between (11–60 m), various measures were observed from pH, Dissolved oxygen (DO), HCO_3_, SO_4_, NH_4_, and NO_3_ relationships versus depth in (m). Alkaline character (pH > 8) was observed for groundwater samples along the depth with lower dissolved oxygen values (O_2_ < 1 mg/l). Also, increments of concentration were observed for SO_4_ and HCO_3_ with a simultaneous decrease in NO_3_ and DO concentrations along groundwater depth. Nitrate arising from different polluted sources has been shown to have different NO_3_/Cl ratios. Chloride is a good indicator of the presence of sewage because it does not undergo any physical, chemical, or biological transformation (Ding et al. 2015).Untreated effluent from manure/sewage is characterized by low NO_3_/Cl ratios and high Cl values. On the other hand, chemical fertilizers have high NO_3_/Cl ratios with small Cl concentrations (Xia et al. 2017). In this study, NO_3_/Cl ratio varied from 0.014 to 0.092 and 0.0021 to 0.771 in surface and groundwater, respectively, (Fig. 11a). This wide variation enhances a mixture of multiple pollution sources. A further examination of Cl/Na vs. NO_3_/Na binary plot (Fig. 11b) reveals that surface and groundwater systems are mainly affected by sewage/manure inputs which are extensively applied in the agricultural area.

### Nitrate pollution source identification based on δ^15^N values

**Fig. 12:**
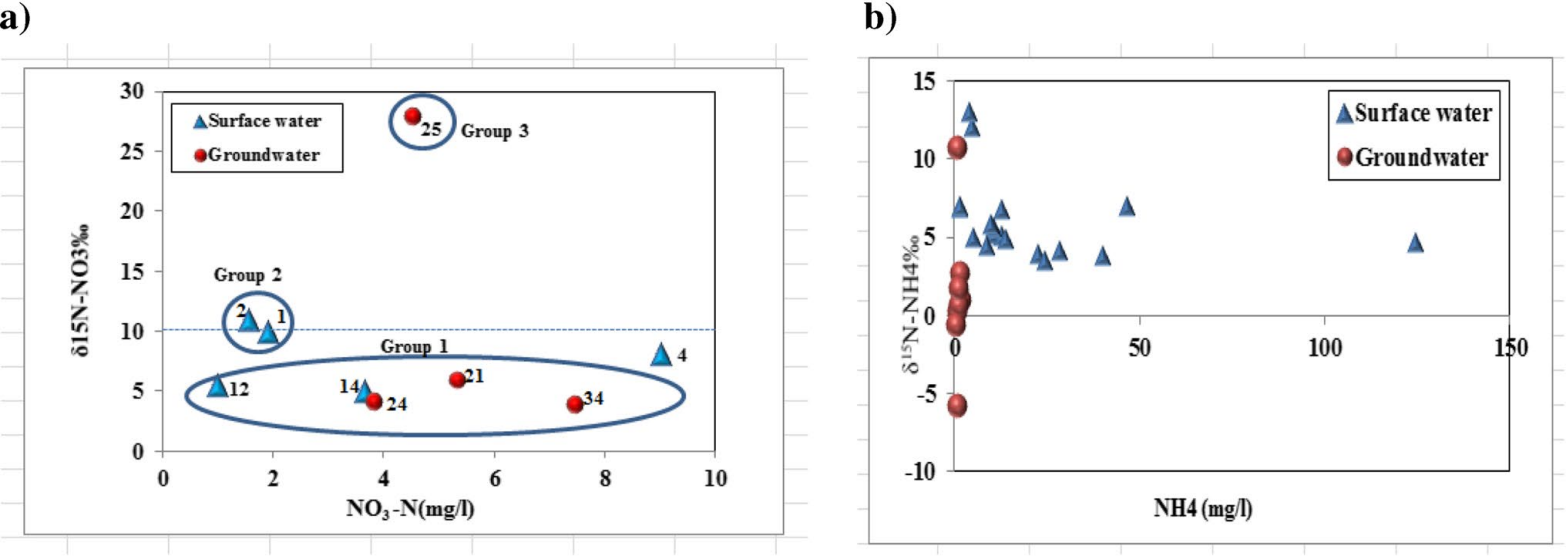
**a** Relationship between NO_3_-N (mg/l) and δ^15^N-NO_3_ in (‰) for collected water samples. **b** Relationship between NH_4_ (mg/l) and δ^15^N-NH_4_^+^ in (‰) for collected water samples

According to several studies, the isotopic signatures of N species (NH_4_^+^ and NO_3_^−^) in surface and groundwater systems under agricultural lands exhibit different ranges depending on N sources availability, transformation processes, and migration pathways (Hosono et al. 2013; Well et al. 2012) that could alter δ^15^N isotopic signature. The results of stable isotope measurement of (δ^15^N-NH_4_^+^ and δ^15^N-NO_3_^−^) in the investigated area are shown in Table 2. From the nitrogen isotope analysis representing replicate sample analysis and the relationship between the isotopic compositions (δ^15^N-NO_3_^−^) vs. NO_3_-N and (δ^15^N-NH_4_^+^) vs. NH_4_^+^ in mg/l concentrations Fig. 12a, b of surface and groundwater samples Fig. 12a can be classified into three groups:

The first group contains 6 samples according to the origin of nitrate pollution. Five samples are located in the north-western part of the study area and have δ^15^N-NO_3_ values ranging from + 3.93 to + 5.94 ‰. The last sample (no. 4) is located at Nahia drain and has δ^15^N = 8.1 ‰. These relatively low δ^15^N values are indicative of mixing between several nitrate sources with different concentrations and isotope values. These mixed sources that yield lower nitrate concentrations could be commercial fertilizer (urea/phosphate) or soil nitrate having δ^15^N values − 6 to + 6‰ and 0 to + 8‰, respectively, which is more pronounced in the northwestern part (surface water samples no. 12, 14 and groundwater sample nos. 21, 24, and 34). Where soil N can occur as organic N plants through atmospheric fixation which with transformation into nitrate leads to acidification:

$$\mathrm N\;\mathrm{from}\;\mathrm{biomass}+\mathrm{soil}\;\mathrm{gas}\leftrightarrow{\mathrm{NH}}_{4+}+2{\mathrm O}_2\rightarrow{\mathrm{NO}}_{3-}+{\mathrm H}_2\mathrm O+2\mathrm H^+$$

This drop in pH can be buffered by calcite dissolution in the soil and /or aquifer as shown in Table 1. For calcite saturation index and dissolved oxygen values where oxic conditions are required to perform this transformation. Indications for the origin of nitrate from biomass in the unsaturated zone were illustrated in soil nitrate δ^15^N values in Table 2 which range from + 5.75 to + 12.85‰ due to the natural accumulation process in the soil. These results are in agreement with natural soil background nitrate concentrations that ranged from 1.34 to 109.2 mg/g NO_3_. Commercial fertilizers such as urea (CO (NH_2_)_2_) are hydrolyzed by urease enzyme and are subsequently converted to nitrate in the unsaturated zone that leached into the groundwater:

$$\begin{array}{c}\mathrm{CO}({\mathrm{NH}}_2)2+\mathrm H^++2{\mathrm H}_2\mathrm O\rightarrow2{\mathrm{NH}}_{4^+}+{\mathrm{HCO}}_{3^-}\\\mathrm{and}\;{\mathrm{NH}}_{4^+}+2{\mathrm O}_2\rightarrow{\mathrm{NO}}_{3^-}+{\mathrm H}_2\mathrm O+2\mathrm H^+\end{array}$$

Urea hydrolysis produces a temporary rise in pH that supports the formation of ammonium NH_3_ that is easily lost to the atmosphere depending on the redox conditions. NH_4_^+^ may be found in groundwater in small concentrations ranging between 0.21 and 1.75 mg/l NH_4_^+^ as shown in Table 2. Group 2 comprise two surface water samples (nos. 1, 2) located in the southern part of the study area at Maruotia Canal, upstream and downstream of El-Moheet Drain having + 10.04‰ and 11.02‰ for δ^15^N-NO_3_ values with 8.43 and 6.85 mg/l NO_3_^−^, respectively. Sources responsible for high nitrate concentrations appear to be affected by sewage or manure with δ^15^N > 10‰ and these effects were pronounced in Fig. 11 showing the binary plots of NO_3_/Cl vs. Cl concentration of the collected water samples. The final group (group 3) comprises one groundwater sample (no.25) located beside El Mansouria Drain (no.10) having δ^15^N- NO_3_ value of 27.91‰ and nitrate concentration of 19.97 mg/l at a shallow depth of 18 m. This sample may be affected by a local source of human activity such as septic tanks. For ammonia concentrations vs. δ^15^N-NH_4_^+^ in (‰) as shown in Fig. 12b, δ^15^N- NH_4_^+^ values of surface and groundwater samples ranged from + 3.5 to 13.01‰ and − 5.7 to 10.8‰, respectively. NH_4_^+^ is one of the major components in surface and groundwater contamination plumes that originate from septic effluents or wastewater released from treatment plants. In untreated sewage, the isotopic signature of δ^15^N- NH_4_^+^ is typically between + 5 and + 9‰ (Cole et al. 2006). This was in agreement with surface water samples (El Moheet drain-Zenin drain- El Rahawy drain and Nahia drain) that receive huge amounts of contaminated sewage water and industrial wastewater containing a high concentration of ammonia, heavy metals and coliform bacteria from different governorates, This source (sewage water) may be mixed with other depleted source such as NH_4_^+^ fertilizers that usually have low δ^15^N values from + 2.7 to + 5.1‰ (Li et al. 2007) where fertilizers, manure, and sewage effluent are the principal anthropogenic sources of NH_4_^+^ pollution under agricultural areas.

## Model simulation results

After model calibration in the steady state, two stress scenarios (transient/unsteady state) on the system are applied.

- *Base case scenario* (pumping rate = 100 m^3^/h) with inflow rate of contaminant (NH_4_^+^ or NO_3_^−^) = 50 m^3^/h.
- *Scenario 1* of (pumping rate = 150 m^3^/h) with inflow rate of contaminant (NH_4_^+^ or NO_3_^−^) = 100 m^3^/h.

**Fig. 13:**
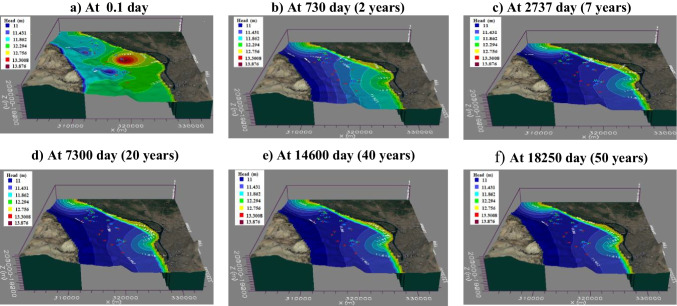
Hydraulic head fluctuation in (m) with time

To simulate and predict the fate and transport behavior of NH_4_^+^ and NO_3_^−^ contaminants in Quaternary aquifer considering advection- dispersion processes and prediction of this transport for 50 years later. Results of water level variation with time and MT3D model for NH_4_^+^ and NO_3_^−^ transport at selected interval times were illustrated as follow: fluctuation in the hydraulic head was observed along the simulation time influenced by groundwater pumping rate as in Fig. 13 where flow direction was directed from northeastern part to southwestern direction with 0.34 m/day groundwater velocities.

**Fig. 14:**
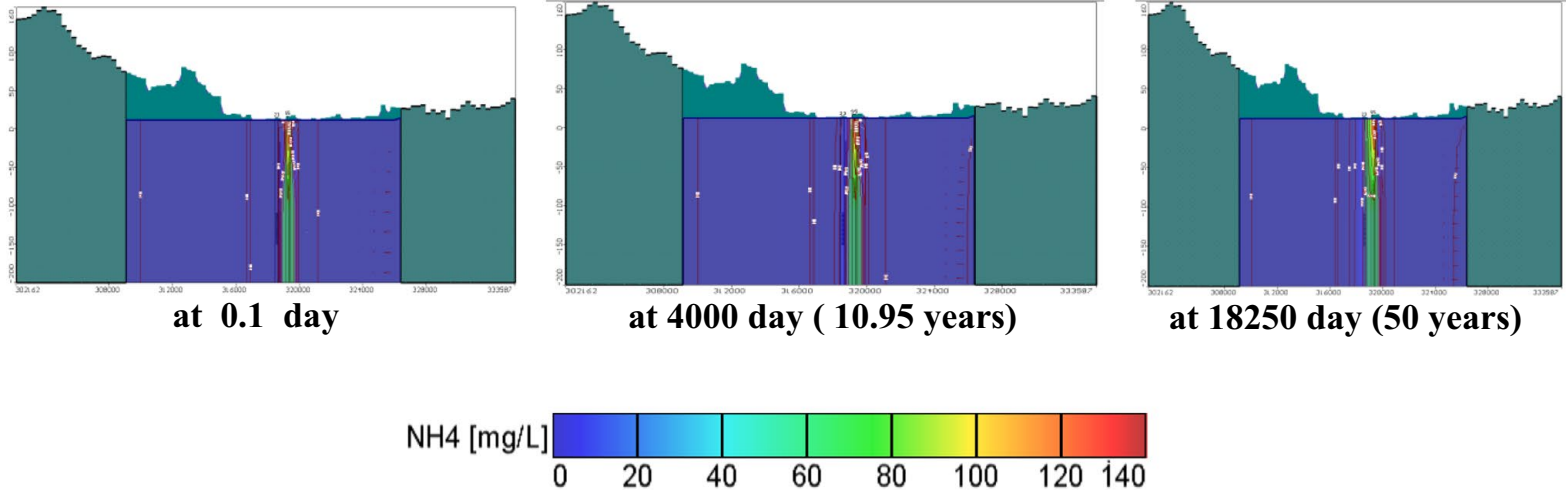
Cross section of NH_4_^+^ concentration using MT3D model results with time

**Fig. 15:**
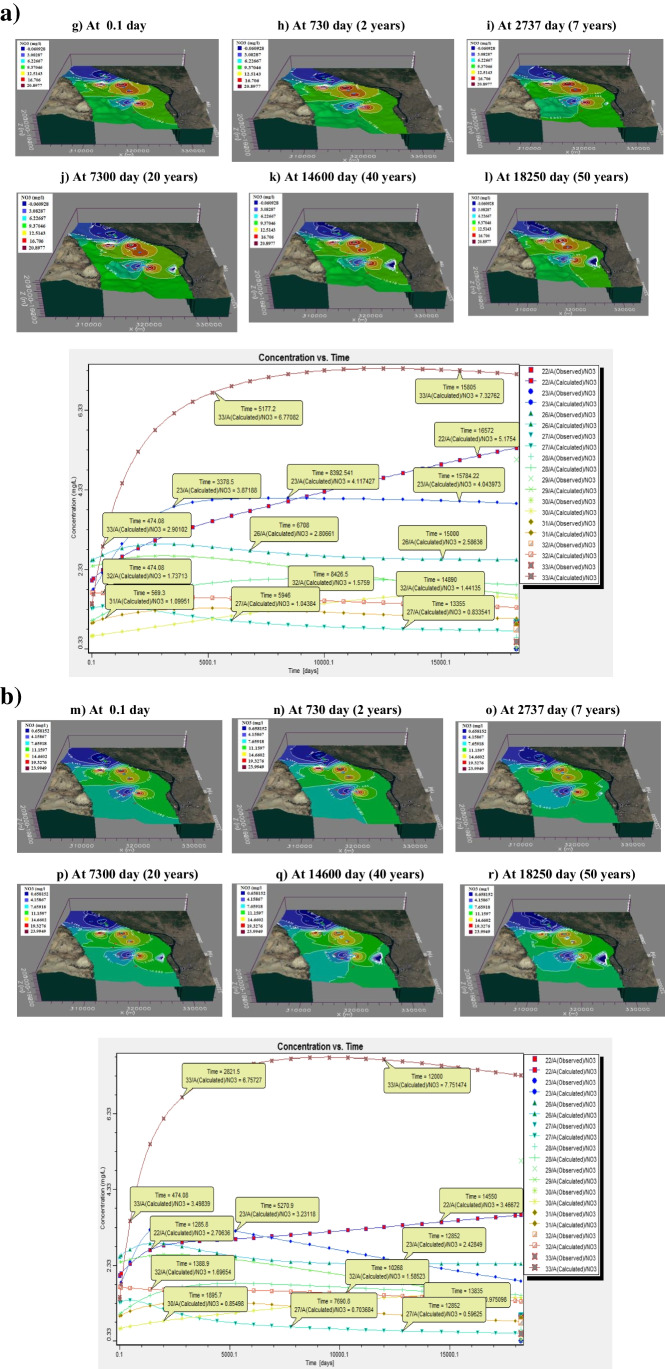
(**a**) Calculated NO_3_.^−^ concentration using MT3D model results with time (base-case scenario). (**b**) Calculated NO3.− concentration using MT3D model results with time (scenario 1)

Results of contaminant transport of NH_4_^+^ and NO_3_^−^ are shown in Figs. 14 and 15, indicating that NH_4_^+^ concentration nearly has no change during the simulation may be due to its high affinity to be sorbed on clay lenses surfaces in the aquifer matrix at PH = 7. For NO_3_^−^, contaminant fluctuations in concentrations depend on many factors (pH, redox potential, chemical/physical parameters, and hydrological structure of the system) (Figs. 15 (a,b)).

Some observations were noticed after applying the stress scenarios for NO_3_^−^ concentration plume using MT3D model results with time as follows:

- Some hand-dug wells have an increment in NO_3_^−^ concentration after a long time of simulation period may be due to agricultural activities and population growth in these rural areas.
- Decreasing of NO_3_^−^ concentration for some hand-dug wells may be due to natural physical–chemical processes that attenuate this concentration during the simulation period.
- Increasing pumping rates are one of the more important factors that lead to an increment of NO_3_^−^ concentration annually which is also connected to land-use activities.
- Local contaminated zones are observed around unlined drains and near wastewater treatment plants (Zenin WWT).
- Simulation for more prolonged times may affect significantly NO_3_^−^ concentration and will be no longer suitable for drinking water purposes.

## Conclusion

The study area located in the southwestern Nile Delta suffers from many environmental problems affecting surface and groundwater systems. The main objective of this study is to investigate surface and groundwater hydrochemistry and distinguish between different sources of ammonia /nitrate pollution using environmental isotopes in addition to tracing the contaminant transport problems of ammonia/nitrate by applying MODFLOW-MT3D model. Representative surface water samples (20 canals and drainage water) and 14 groundwater samples were collected and analyzed for physical/chemical and stable isotope measurements (δ^18^O, δD, δ^15^N_NH4_, and δ^15^N_NO3_). Total dissolved solids (TDS) for surface water ranged from 256 to 2451 mg/l with a mean value of 809 mg/l exceeding WHO limits of 1000 mg/l for only 15% of the collected surface water samples. However, groundwater samples exceeded WHO limits by 71% with TDS values ranging from 530 to 2410 mg/l and a mean value of 1342 mg/l. Trace elements analysis results for collected water samples illustrated that Pb, Cd, and Ni have higher concentration values compared with Maximum concentrations levels of trace elements for drinking water. Stable isotope composition of δ^18^O and δD for surface and groundwater samples have enriched values which suggest the evaporation process affects the collected water samples and irrigation return water that contributes to aquifer recharge. Based on ammonia and nitrate concentrations and their nitrogen isotope values, three main sources, sewage water, fertilizers, and soil nitrogen, were found to be responsible for water system impairment.

## Recommendations (suggested solutions)

This research gives some recommendations to be undertaken to attenuate this risk as follows:

- As this study determined sewage water as the main source of ammonia/nitrate pollution in the study area, a pretreatment of this wastewater with the establishment of sewage network was necessary in addition to continuous monitoring of contaminants concentrations especially ammonia/nitrate in surface and groundwater systems.
- The results of this study have confirmed that the comprehensive application of the methods mentioned before is effective tools to decipher local nitrogen contamination of groundwater and delineate the potential transport pathways of N pollutants over a long-term period. Additionally, it can support the water resources management and decision makers for local authorities to protect groundwater quality, reduce human health risks and provide sustainable clean drinking water through making practical decisions.
- Applying multidisciplinary approaches including hydrogeochemistry, multiple environmental stable isotopes (δ^2^H, δ^18^O, δ^15^N-NH_4_, and δ^15^N-NO_3_) coupled with nitrogen transport numerical modeling (MODFLOW-MT3D) were not sufficient to identify origins and processes influencing nitrate sources in surface and groundwater systems for the unconfined aquifer that suffering from severe human activities. More steps could be done may be by measuring δ^18^O-NO_3_ and some chemical markers (CEC compounds) of fecal contamination to differentiate closely related sources of nitrate contamination such as sewage and manure in future research work.

## Data Availability

All data generated or analyzed during this study are available and included in this manuscript.
